# Single patch super-resolution of histopathology whole slide images: a comparative study

**DOI:** 10.1117/1.JMI.10.1.017501

**Published:** 2023-01-31

**Authors:** Mehdi Afshari, Saba Yasir, Gary L. Keeney, Rafael E. Jimenez, Joaquin J. Garcia, Hamid R. Tizhoosh

**Affiliations:** a University of Waterloo, Kimia Lab, Waterloo, Ontario, Canada; b Mayo Clinic, Anatomic Pathology, Rochester, Minnesota, United States; c Mayo Clinic, Artificial Intelligence and Informatics, Rochester, Minnesota, United States

**Keywords:** histopathology, super-resolution, deep neural networks, generative adversarial networks

## Abstract

**Purpose:**

The latest generation of scanners can digitize histopathology glass slides for computerized image analysis. These images contain valuable information for diagnostic and prognostic purposes. Consequently, the availability of high digital magnifications like 20× and 40× is commonly expected in scanning the slides. Thus, the image acquisition typically generates gigapixel high-resolution images, times as large as 100,000×100,000  pixels. Naturally, the storage and processing of such huge files may be subject to severe computational bottlenecks. As a result, the need for techniques that can operate on lower magnification levels but produce results on par with outcomes for high magnification levels is becoming urgent.

**Approach:**

Over the past decade, the digital solution of enhancing images resolution has been addressed by the concept of super resolution (SR). In addition, deep learning has offered state-of-the-art results for increasing the image resolution after acquisition. In this study, multiple deep learning networks designed for image SR are trained and assessed for the histopathology domain.

**Results:**

We report quantitative and qualitative comparisons of the results using publicly available cancer images to shed light on the benefits and challenges of deep learning for extrapolating image resolution in histopathology. Three pathologists evaluated the results to assess the quality and diagnostic value of generated SR images.

**Conclusions:**

Pixel-level information, including structures and textures in histopathology images, are learnable by deep networks; hence improving the resolution quantity of scanned slides is possible by training appropriate networks. Different SR networks may perform best for various cancer sites and subtypes.

## 1 Introduction

Histopathology investigates tissue specimens traditionally employing light microscopy. However, recent technological advancements have empowered pathologists to transit to digital pathology to use high-resolution (HR) scanning and storage of glass slides. The generation of whole slide images (WSIs) in digital pathology accelerates the research and clinical utility of computerized techniques. Among other advantages, digital pathology enables the inspection and comparison of tissue samples with annotated digital images. Available metadata may include clinical data, such as pathology reports and molecular data.

One of the main objectives in digital pathology is investigating image analysis applications. Computer-assisted diagnosis (CAD) of histopathology images is practical due to repetitive tissue patterns that automated recognition can exploit.^1^ Furthermore, as a relatively new technology, WSI allows assessing various techniques to analyze HR images. However, a series of problems, such as high demand for storage, labelling (annotating) image regions, and scanning quality are still hindering the practical usage of digital pathology.^2^ Overcoming these challenges may improve the diagnosis by correlating comparable patients and retrieving similar images through “image search.”

During the past few years, microscopic images were in the focus of many researchers in deep learning.^3^^,^^4^ Overall, proposed approaches mainly concentrate on the classification of WSI patches resulting from sliding a window.^5^ Generally, CAD algorithms require HR images. Diagnosis in histopathology relies on the information of the tissue samples mounted and fixed on glass slides visually inspected at several magnification levels. It is common for the pathologist to repeatedly zoom in and out on specific tissue regions to locate and categorize abnormalities. Consequently, we digitize glass slides at high magnifications to generate HR images for computational pathology. The gigapixel WSIs are extremely large files [usually in scanscope virtual slide (SVS) or BigTiff format] that inevitably lead to high storage needs and transfer bottlenecks. Each scan is generally an image of gigabyte size, and for each patient, multiple glass slides are usually available. Every day, numerous patients are biopsied. All these factors result in the creation of massive histopathology archives that may not be easily accessed or analyzed for research and clinical purposes. Hospitals and clinics may decide to erase older scans to free up some storage space, which is a rather undesirable solution leading to loss of valuable information in evidently diagnosed cases. Instead of removing older slides, a more desirable solution is keeping a low-resolution (LR) version of slides and upsampling it to HRsupon access, with no information loss.

In contrast, digital scanners are expensive devices that may not be available to all pathologists worldwide. However, the availability of lower-cost devices may be more feasible but would negatively impact the scanning resolution. Having tools to virtually increase the WSI resolution at any magnification of scanned slides by affordable devices facilitates the adoption of digital pathology, hence enabling computational pathology.

Finally, the scans and the scanners are far from ideal. Digitized slides may be blurry in some of the regions of a scan. This issue is not rare since the optical devices of the scanner need to focus on the biopsy. The digital scanners manage the proper focusing by measuring the depth of the tissue surface. However, focusing is error-prone due to issues like specks of dirt or stains on a slide and physical limitations of optics.

All in all, some notion of upsampling of the scanned images may help solve these problems. This study mainly aims to explore available methods capable of upsampling WSIs. The overall approach would help with storing smaller images while capable of restoring HR upon request. The LR images are magnifiable by new types of decompression methods.

A promising approach to address the discussed issues is super-resolution (SR). In this technique, the goal is to retrieve pixel-level information of an image based on perceptual information. Beyond images, some researchers work on video resolution enhancement in computer vision using SR.^6^ Generally, SR is implemented using classical methods like kernel-based techniques.^7^ However, recent deep-learning approaches outperform traditional methods by a such large margin that it is quite difficult to find reports that compare the two. The SR technique has intertwined with deep neural networks during the past few years.^8^

Image SR is a class of image-processing techniques aiming to construct a HR image based on a LR image. Deep learning has recently successfully addressed several image enhancement problems in computer vision. The single image SR (SISR) network uses a single LR image to reconstruct its HR mapping. The essence of the problem has been approached in multiple real-world applications, including but not limited to medical imaging^9^^–^^12^ and security.^13^^–^^15^ In addition, image SR improves the performance of other tasks.^16^^–^^18^ Image SR is a considerably challenging issue which also is an ill-posed problem due to its intrinsic problem definition. A single LR image can act as the respective LR image of multiple HR images. Conventional techniques emerged a few decades ago.^19^ Among the classical methods, researchers introduced methods based on predictions,^20^ edges,^21^ statistics,^22^ patches,^23^ and sparse representation.^24^

Deep learning has largely impacted image SR. In this area, state-of-art results have been achieved recently. Specific benchmarks for this task are also designed but these are commonly general-purpose images.^25^^–^^28^ Deep networks to address this problem stem from the very early convolutional neural networks (CNNs), such as SRCNN,^29^ to more recent ideas like generative adversarial networks (GANs), which have been employed in SRGAN.^30^

Deep SR networks differ in aspects such as the architecture of the network^31^^–^^33^ loss function^34^^,^^35^ and learning strategies^36^^,^^37^.

Many researchers have also applied SR in medical imaging. There are publicly available datasets to train networks for SR tasks. However, the introduced SR datasets rarely focus on the domain of histopathology. Subsequently, there are only a few SR studies in histopathology.

Medical imaging contains many image modalities for diagnostic and treatment-panning purposes. Researchers initially investigated classical SR methods on radiological modalities like magnetic resonance imaging. Radiology images are generally small (mainly in megapixel range) and hence easier to process.^38^ However, by the emergence of deep learning and better results, more complex structures and data like pathology images are being processed. In this study, the main focus is on histopathology images.

Among the earliest SR in histopathology studies was a paper published by Mukherjee et al. in 2018. The authors introduced a deep framework for reconstructing HR images in the pathology. Their results showed promising outcomes, which they suggested as a comparative counter-part to the expensive scanners.^39^ Later, they investigated a recurrent network to enhance the quality by a multiresolution approach. Lately, Bin Li et al. proposed a framework to benefit from the hierarchical structure of the WSIs and achieved good results. Their approach showed that downsampling could act as a training data solution for these deep networks. The studies used tissue microarray datasets and a two-site whole tissue section dataset.^40^

This study is structured as follows. First, the general SR problem, which corresponds to the upsampling method, is described. This section briefly elaborates on the commonly used traditional upsampling methods. Next, in Sec. 3, deep SR networks are discussed, and common aspects like architecture, loss function, and assessment metrics are described. Then the experiments and training phase discussions are presented. Next, in Sec. 4, the materials used for the experimentation are described. Finally, in Sec. 5, the results and conclusion summarize the outcomes.

## 2 Problem Formulation

The main goal of SR is to find the most relevant HR image that corresponds to a LR image $I_{\mathrm{LR}}$. We assume that the LR image is an image of $l_{h}\times l_{v}$ pixels (where $l_{h}$ and $l_{v}$ are the number of pixels in horizontal and vertical axis, respectively); therefore, LR image consists of $s_{\mathrm{LR}}=l_{h}\times l_{v}$ pixels and $s_{\mathrm{HR}}=m\times s_{\mathrm{LR}}$ is the number of pixels of the HR image $I_{\mathrm{HR}}$. The integer parameter m is the factor that shows the increase of the image size. Now, the degradation is defined as

$$I_{\mathrm{LR}}=D(I_{\mathrm{HR}};\delta ).$$ (1)

Here D is the degradation function and δ corresponds to the related parameters (e.g., kernel size, noise, and scaling factor). The degradation function is assumed to be unknown in many problems called blind SR; however, we can consider it known if the degradation is digitally applied. The approximate HR image $I_{\mathrm{SR}}$, which is called the SR image of the LR image, is then constructed according to

$$I_{\mathrm{SR}}=F(I_{\mathrm{LR}};\theta ).$$ (2)

Here F and θ correspond to the SR function and the parameters of the approximation, respectively. The degradation model could be modeled by a downsampling function applied to the HR image

$$D(I_{\mathrm{HR}};\delta )=(I_{\mathrm{HR}}){↓}_{d},\{d\}\subset \delta ,$$ (3)

where ${↓}_{d}$ denotes the downsampling operation and the set {d} are the parameters of it. Many downsampling methods are introduced in the literature.^41^^,^^42^ Among these methods, bicubic downsampling is commonly used in image SR applications, although other methods are used as well. The bicubic upsampling method has been covered in Sec. 2.1. Generally, it is possible to model the downsampling as^43^

$$D(I_{\mathrm{HR}};\delta )=(I_{\mathrm{HR}}⊗k){↓}_{d}+n_{\zeta },\{k,d,\zeta \}\subset \delta .$$ (4)

Here $I_{\mathrm{HR}}⊗k$ denotes a kernel of k convolution with the HR image to apply a filter (e.g., blurring) on the image. $n_{\zeta }$ is the Gaussian noise added to the model with an average of zero and the standard deviation of ζ. The model described by Eq. (4) has been proven to have more relativity with real-world problems.^44^

Finally, the image SR objective is formulated as

θ^=arg minθ L(Isr,Ihr)+λΦ(θ). (5)

Here, $L(I_{\mathrm{SR}},I_{\mathrm{HR}})$ is the loss function which is measuring the difference between the generated SR image and the ground truth HR image. The regularization term is formulated by Φ(θ), and λ is the trade-off parameter. The losses are usually a combination of multiple functions.

### 2.1 Interpolation

**Fig. 1:**
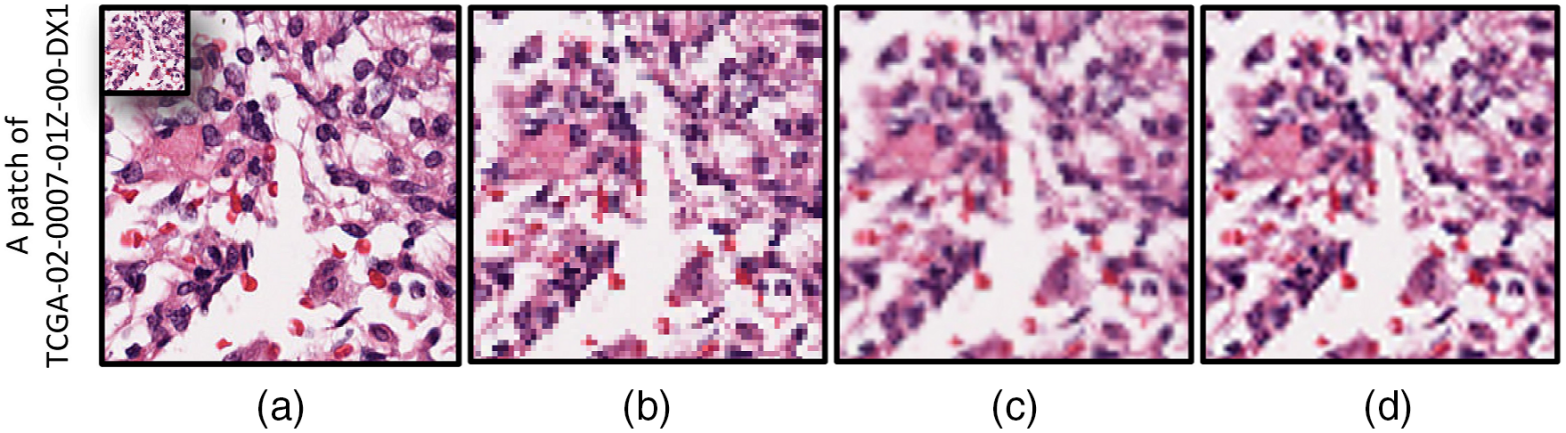
A TCGA brain patch of the size 256×256 pixels and the interpolation of its downsampled patch of the size 64×64 pixels: (a) original image and the downsampled image in top right, (b) nearest neighbor interpolation, (c) bilinear interpolation, and (d) bicubic interpolation.

Interpolation of the data is a part of either upsampling or downsampling. Here, we briefly explore the upsampling methods while the downsampling can be analogously described. In image resampling, the main aim is to predict pixel values based on the available data. This task has been conventionally investigated to introduce relatively fast and easy methods. Some of these methods are the nearest neighbor, bilinear, and bicubic interpolations. They aim to construct a smoother image. Sample images for these methods are shown in Fig. 1.^45^ The nearest neighbor methods assign the closest available value of known pixels to the unknown ones. The bilinear approach estimates the value of unknown pixels by constructing a bilinear in x and y axis directions, while the bicubic implements the same idea but in a second-order function.

## 3 SR Deep Networks

During the past few years, SISR, like other areas, has significantly improved. These improvements were not possible without deep neural networks. Wang et al. categorized the deep neural networks for SR into four main types, which have been summarized in following section. ^43^ The categories with samples of the successful networks are discussed in Sec. 3.1. Then the loss function and evaluation metrics are given in Secs. 3.2 and 3.3, respectively.

### 3.1 Architectures

One of the first networks for image SR is SRCNN, which Dong et al. introduced in 2014. The network they introduced enhanced the classical mapping methods between the LR and HR images. Their success gained attention, so many other networks were proposed based on their initial proposal. A pre-upsampling module follows their proposed CNN network to enhance the image initially. A pre-upsampling strategy enlarges the LR image first and feed it into the network. In contrast, the post-upsampling approach processes the LR at its original size through the layers of the network and then upsample the image. Despite their success, these structures faced issues like noise amplification. Later, researchers shed light on the considerably lengthy operation time of these networks due to the higher dimensional computation framework comparing other networks.^29^

Post-upsampling networks then solve the computational expense problem. Researchers benefitted from similar ideas for shorter training and testing times. Residual channel attention network (RCAN) and SRGAN were among the networks that benefited from this framework. These networks were successful enough to establish state-of-the-art results. Although RCAN presented higher-accuracy values in many studies, SRGAN presented more realistic images by hallucinating the textures.^30^^,^^46^

The benefits of the postupsampling networks were promising; however, there were also shortcomings. The single-step upsampling module made the training an arduous task when a higher magnification like 4 or 8 was required. To help this issue, researchers that experienced the high-quality results of progressive networks such as StyleGAN implemented similar approaches in the areas of SISR.^47^ One of these networks that were upsampling iteratively is ProSR which achieved relatively high performance.^37^

Finally, iterative up-and-down sampling SR was investigated as well since even the progressive upsampling encountered some problems. For instance, the learning strategies required to train them were rather complicated. In contrast, iterative upsampling applies back-projection refinement. To name a successful example, feedback network for image SR (SRFBN) fits this category which benefits from feedback block and offers better representations.^48^

### 3.2 Loss Functions

In deep learning, loss functions are crucial in guiding the model to optimize with desired weights. In image SR, the optimal outcome is a network that enables a high-quality reconstruction. Therefore, the loss functions are designed to help the training lead the network to reconstruct an image close to the HR image. Here, we discuss three of the loss functionals, which are used in many studies.

#### 3.2.1 Reconstruction loss

Reconstruction loss or pixel loss indicates the similarity of the generated SR image and the desired HR image. This is computed by L1-norm or L2-norm of the image differences as

$$L_{\mathrm{rec}.}(I_{\mathrm{HR}},I_{\mathrm{SR}})=\frac{1}{v}{\left\|I_{\mathrm{HR}}-I_{\mathrm{SR}}\right\|}_{1},$$ (6)

$$L_{\mathrm{rec}.}(I_{\mathrm{HR}},I_{\mathrm{SR}})=\frac{1}{v}{\left\|I_{\mathrm{HR}}-I_{\mathrm{SR}}\right\|}_{2}.$$ (7)

where v=h×w×c is the volume which is the multiplication of height and weight and number of channels. $L_{1}$-norm showed better performance from sharpness in addition to easier convergence. The sharper images emerge since the $L_{2}$-norm penalizes the significant deviation exceedingly while minor errors slightly. It is important to note that $L_{0}$ for image comparison corresponds to the pixel equality where it cannot backpropagate meaningful information during learning. Thus, the use of $L_{0}$ norm alone if not facilitated by other methods is usually not practiced.

#### 3.2.2 Perceptual loss

A perceptual loss, also called content loss, evaluates the perceptual similarity of the generated image with the HR image. This is done by comparing the semantic content of the images using a pretrained network (e.g., VGG16). Researchers computed the distance of the l’th layer of the network based on

$$L_{\mathrm{percept}.}(I_{\mathrm{HR}},I_{\mathrm{SR}})=\frac{1}{v}{\left\|\phi^{\left(l\right)}(I_{\mathrm{HR}})-\phi^{\left(l\right)}(I_{\mathrm{SR}})\right\|}_{2},$$ (8)

where $\phi^{\left(l\right)}(\cdot )$ denotes the output of the l’th layer of the pre-trained deep network. In other words, the perceptual loss guides the network based on the hierarchical image features of a network that has been trained for a task (e.g., ImageNet classification).^49^

#### 3.2.3 Adversarial loss

The application of GANs has reached the SR as well. In general, a GAN-based training consists of two main networks, namely the “generator” and the “discriminator.” These two networks compete against each other. Consequently, the adversarial loss for the generator and discriminator networks, respectively, is defined as

$$L_{{\mathrm{GAN}}_{G}}(I_{\mathrm{HR}};D)=-\log\;D(I_{\mathrm{SR}}),$$ (9)

$$L_{{\mathrm{GAN}}_{D}}(I_{\mathrm{HR}},I_{\mathrm{SR}};D)=-\log\;D(I_{\mathrm{HR}})-\log(1-D(I_{\mathrm{SR}})).$$ (10)

D denotes the discriminator for a binary decision whether the image is real data or generated.

### 3.3 Reconstruction Quality Measurement

Human operators assess image quality most reliably. Although the reliability is ensured by human inspection, the mainstream methods are supposed to computational methods for higher efficiency. Here, we briefly describe two of the most common metrics in this area.

#### 3.3.1 Peak signal-to-noise ratio

The peak signal-to-noise ratio (PSNR), as one of the most commonly used metrics, measures the maximum pixel value rate to the mean squared error of the images. The PSNR of the two images $I_{\mathrm{SR}}$ and $I_{\mathrm{HR}}$ is then computed by

$$\mathrm{PSNR}=10\;\log_{10}(\frac{L^{2}}{{\left\|I_{\mathrm{HR}}-I_{\mathrm{SR}}\right\|}_{2}^{2}}),$$ (11)

where L is the maximum pixel-value which usually is equal to 255. Despite the common use of this metric, it does not reflect the human perception.

#### 3.3.2 Structural similarity

The human visual system is primarily concerned with recognizing image structures. Hence, a structural similarity metric is proposed to extract structural information from images. This metric is a combination of three parts, including luminance, contrast, and structures. It is given as

$$\mathrm{SSIM}=\frac{2\mu_{\mathrm{SR}}\mu_{\mathrm{HR}}+k_{1}}{\mu_{\mathrm{SR}}^{2}+\mu_{\mathrm{HR}}^{2}+k_{1}}\times \frac{\sigma_{\mathrm{SR},\mathrm{HR}}+k_{2}}{\sigma_{\mathrm{SR}}^{2}+\sigma_{\mathrm{HR}}^{2}+k_{2}},$$ (12)

where $\mu_{\mathrm{SR}}$ and $\sigma_{\mathrm{SR}}$ are the mean and variance of the SR image, $\mu_{\mathrm{HR}}$ and $\sigma_{\mathrm{HR}}$ are the mean and variance of the HR image, and $\sigma_{\mathrm{SR},\mathrm{HR}}$ is the covariance between SR and HR image. Finally, $k_{1}$ and $k_{2}$ are the relaxation constants.

### 3.4 Experiments

The reconstruction path uses LR patches, and a deep network trained for SISR generates the patches for synthetic WSI. Six networks are trained to find a better network that can adequately enhance the images. First, we benefit from the deep back-projection networks (DBPNs). The network benefits from iterative up- and downsampling layers called stages. The stages include an error feedback mechanism for projection errors. The network can learn multiple content information by its up-and-downsampling layers.^50^ Residual dense networks (RDNs) are then trained to exploit the hierarchical features from all the convolutional layers.^51^ The third is a very deep residual channel attention network (RCAN) that benefits from residual in residual blocks in a post-upsampling structure.^46^ Forth is an SRFBN that focuses more on feedback mechanisms based on the fact that the human visual system follows a similar method.^48^ Fifth is enhanced deep residual networks for SISR (EDSR), which is proposed in addition to the multi-scale deep SR system.^36^ Finally, a network we use to do the SR is among the enhanced SR GANs (ESRGAN). This network architecture has achieved some state-of-the-art results in addition to providing a high perceptual index.^52^

### 3.5 Training

The training patches of the extracted SR dataset are used to train the network. The relativistic discriminator is used for adversarial training to achieve the best results. The discriminator classifies the images as fake or real and relatively compares the extent of fake and real images that enhance the learning procedure. The networks are all trained for at least 100,000 iterations. The optimal weights selection is on the basis of the validation accuracy and loss. The training were done on a Tesla V100 32 GB GPU.

## 4 Materials

WSIs are the scanned histopathology slides. The cancer genome atlas (TCGA) is the largest publicly available scanned slides and reports dataset. This dataset (available at Ref. 53) allows researchers to experiment and propose new methods and compare their results easily. The generated data by TCGA is now over 2.5 petabytes and spans genomic, epigenomic, transcriptomic, and proteomic data.^54^

TCGA repository (i.e., genomic data commons) contains 11,007 cases and 30,072 SVS files of the slides. The WSIs of this repository span 26 organs (primary sites) with 32 cancer subtypes. The subtypes are abbreviated by a few letters in the repository. Complete subtype names and the distribution of the number of the patients in each category is explained in Table 1. The demographic information attached to each scan includes “morphology,” “primary diagnosis,” “tissue or organ of origin,” “patient age at the time of diagnosis,” “tumor stage,” “age,” “gender,” “race,” and “ethnicity” and some other information like the patients current status (e.g., dead or alive).

**Table 1:** The TCGA codes (in alphabetical order) of all 32 primary diagnoses and corresponding number of evidently diagnosed patients in the dataset.

Code	Primary diagnosis	Number of patients
ACC	Adrenocortical carcinoma	86
BLCA	Bladder urothelial carcinoma	410
BRCA	Breast invasive carcinoma	1097
CESC	Cervical squamous cell carcinoma and endocervical adenocarcinoma	304
CHOL	Cholangiocarcinoma	51
COAD	Colon adenocarcinoma	459
DLBC	Lymphoid neoplasm diffuse large B-cell lymphoma	48
ESCA	Esophageal carcinoma	185
GBM	Glioblastoma multiforme	604
HNSC	Head and neck squamous cell carcinoma	473
KICH	Kidney chromophobe	112
KIRC	Kidney renal clear cell carcinoma	537
KIRP	Kidney renal papillary cell carcinoma	290
LGG	Brain lower grade glioma	513
LIHC	Liver hepatocellular carcinoma	376
LUAD	Lung adenocarcinoma	522
LUSC	Lung squamous cell carcinoma	504
MESO	Mesothelioma	86
OV	Ovarian serous cystadenocarcinoma	590
PAAD	Pancreatic adenocarcinoma	185
PCPG	Pheochromocytoma and paraganglioma	179
PRAD	Prostate adenocarcinoma	499
READ	Rectum adenocarcinoma	170
SARC	Sarcoma	261
SKCM	Skin cutaneous melanoma	469
STAD	Stomach adenocarcinoma	442
TGCT	Testicular germ cell tumors	150
THCA	Thyroid carcinoma	507
THYM	Thymoma	124
UCEC	Uterine corpus endometrial carcinoma	558
UCS	Uterine carcinosarcoma	57
UVM	Uveal melanoma	80

### 4.1 Creation of a Dataset

This section describes the creation of a dataset of histopathological slides. The dataset includes patches, and the labels of the patches are the information attached to the WSIs. The scanned slides of the TCGA repository include many frozen section WSIs. A frozen section (cryosection) is a laboratory technique that helps to get to microscopic analysis of a specimen rapidly. The fast diagnosis is beneficial for the management of the patient during an operation. However, due to its procedure, frozen sections are not usually of high quality. These lower-quality slides were dropped to avoid confusion for deep network training sessions. Frozen section scans and diagnostic slides do not belong to the same domain from a machine-vision perspective. As well, frozen sections are prepared using a different, more rapid process. The image SR problem seeks to translate one instance within the same domain to another instance. Therefore, for a proper application, the images in each must be from the same domain. Hence, we only used diagnostic TCGA slides.

To assign labels to the images, the information provided by the repository are used. The beneficial information is determined to be the primary diagnosis and the section site (/tissue/organ of origin). These two labels are presented under the “diagnoses/treatments” of the slides. Although other information are also available for further diagnosis, we have decided to use the aforementioned labels.

Finally, some of the slides did not include the required information to create the image labels. These slides were removed. In addition, the classes with a number of WSIs <20 were also eliminated.

### 4.2 Dataset for Histopathology SR

**Fig. 2:**
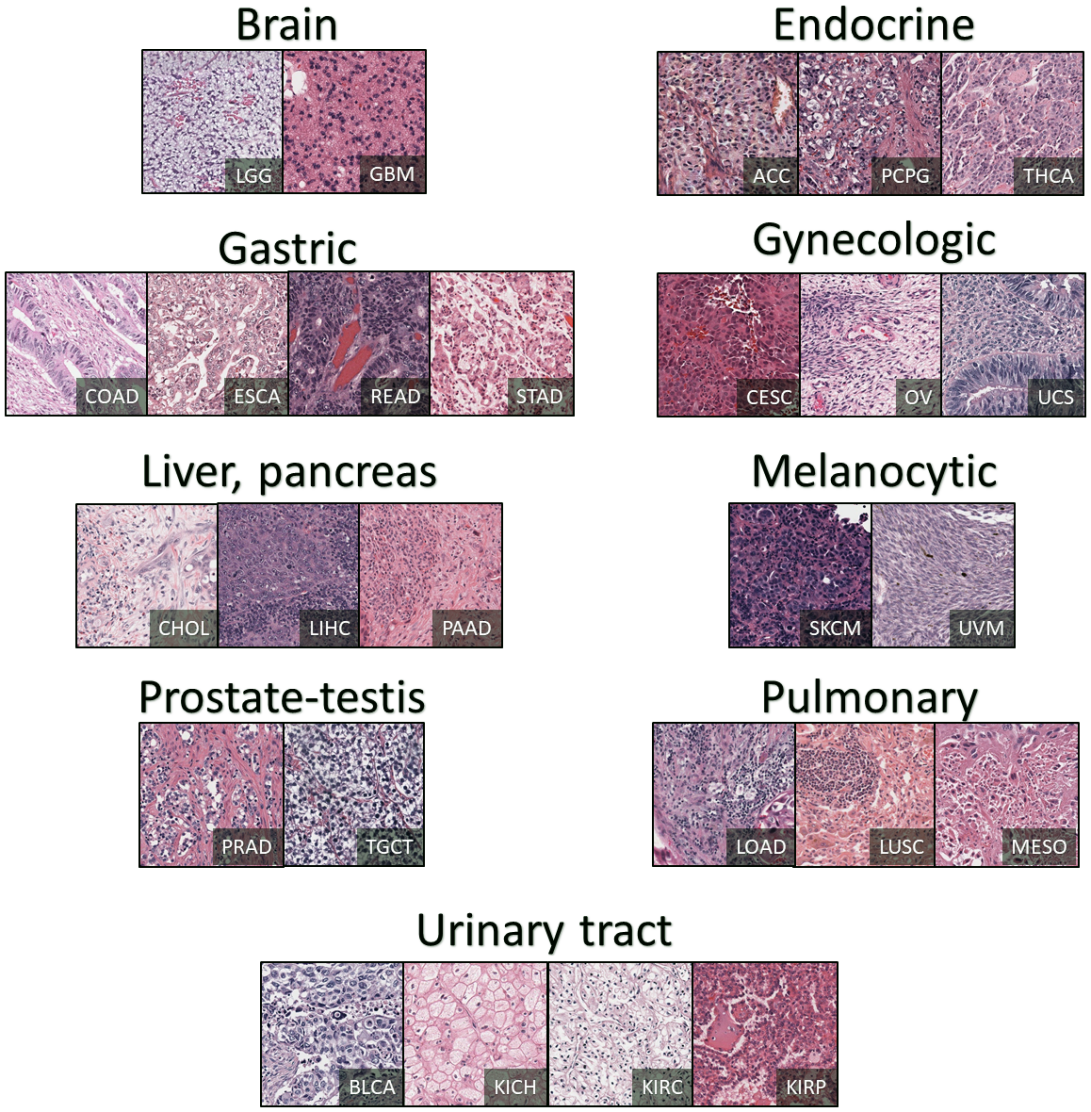
Patches of the created SR dataset in nine TCGA cancer subtypes.

To create a dataset for histopathology SR, some considerations may be necessary to eliminate the appearance of undesired data. We have first removed the background patches to minimize the artifact since these do not contain valuable tissue information for training. Second, the slides are cropped into the same size tiles. The tiles (or patches) are 640×640, 320×320, 160×160, 80×80, and 16×16  pixels in three channels of RGB at 40×, 20×, 10×, 5×, and 1× magnification levels. One sample per cancer subtype is provided in Fig. 2.

### 4.3 Diagnostic Dataset

The breast cancer histopathological image classification (BreaKHis) dataset was utilized to evaluate the quality and diagnostic accuracy of the SR images compared to original magnifications.^55^ The dataset consists of 9109 microscopic pictures of breast tumor tissues obtained from 82 individuals at four magnification levels. The primary categories of the BreaKHis dataset are benign and malignant tumors. There are a total of 1820 pictures at 400× magnification, of which 588 are malignant and 1232 are benign. The images are 700×460  pixels in size, 3-channel RGB with 8-bit depth per channel, and saved in PNG format.

## 5 Results and Discussions

The results are assessed from multiple perspectives. First, we provide the quantitative comparison of trained networks results for image generation based on PSNR and structural similarity index measure (SSIM) in Table 2. These values are expected to quantify similarity at some level within the same tissue type. For instance, although the fatty content may contribute to variance in these values, the anatomic structure of tissue in various organs is not same. Among others, differences in these values may stem from various cell density and size in different tissue types. In this table, we first compare different sites and subtypes against each other. The comparison shows the details and complexity of the images in each category. The minimum and maximum reported values of the 4× bicubic upsampling of the LR images is shown in italic and bold italic, respectively. It is evident that brain/glioblastoma multiforme (GBM) has the lowest SSIM, which could be interpreted as the highest structural loss within a traditional downsample upsample procedure. In contrast, the endocrine/thyroid carcinoma (THCA) offers the highest SSIM value. This could be interpreted as the maximum complexity of sharp structures and minimum, respectively. In contrast, the lowest and highest PSNR values were observed in testis/testicular germ cell tumors (TGCTs) and liver cholangiocarcinoma (CHOL). These values show the extent of details lost in the interpolation.

**Table 2:** Accuracy of image reconstruction using six networks trained on TCGA dataset; the PSNR/SSIM numbers are shown where the bold numbers are best reconstructions across all networks; and italic and bold italic are the worst and best bicubic reconstruction among data categories, respectively.

Data categorization		Accuracy (PSNR/SSIM)						
Site	Subtype	Bicubic	DBPN	RDN	RCAN	SRFBN	EDSR	ESRGAN
Brain	GBM	*(23.46/0.57)*	**(24.64/0.66)**	(24.44/0.66)	(24.44/0.66)	(23.68/0.62)	(23.99/0.63)	(22.43/0.57)
	LGG	(23.50/0.58)	**(24.68/0.67)**	(24.50/0.67)	(24.50/0.67)	(23.69/0.63)	(24.02/0.64)	(22.35/0.58)
Endocrine	ACC	(22.30/0.59)	(23.52/0.68)	(18.68/0.43)	**(23.55/0.69)**	(22.64/0.65)	(22.81/0.64)	(21.85/0.63)
	PCPG	(22.17/0.59)	(23.42/0.68)	(18.39/0.41)	**(23.52/0.70)**	(22.47/0.65)	(22.65/0.64)	(21.73/0.63)
	THCA	***(24.30/0.67)***	(25.78/0.75)	(19.02/0.39)	**(25.96/0.76)**	(25.12/0.73)	(24.75/0.70)	(24.21/0.72)
Gastrointestinal	COAD	(23.56/0.62)	**(24.92/0.71)**	(18.80/0.40)	(24.90/0.72)	(24.14/0.69)	(24.00/0.67)	(23.26/0.67)
	ESCA	(23.52/0.65)	(25.17/0.74)	(18.83/0.42)	**(25.17/0.75)**	(24.14/0.71)	(24.13/0.70)	(23.46/0.70)
	READ	(23.38/0.59)	(24.61/0.68)	(19.13/0.38)	**(24.66/0.69)**	(23.97/0.66)	(23.82/0.64)	(23.00/0.63)
	STAD	(23.07/0.64)	**(24.56/0.73)**	(18.22/0.41)	(24.56/0.74)	(23.68/0.70)	(23.61/0.69)	(22.98/0.68)
Gynecologic	CESC	(22.75/0.62)	(24.26/0.71)	(18.13/0.40)	**(24.31/0.72)**	(23.45/0.69)	(23.22/0.66)	(22.56/0.66)
	OV	(23.25/0.59)	(24.45/0.67)	(19.22/0.40)	**(24.53/0.69)**	(23.90/0.66)	(23.75/0.64)	(23.11/0.64)
	UCS	(23.02/0.62)	(24.48/0.71)	(18.43/0.41)	**(24.50/0.72)**	(23.68/0.69)	(23.44/0.66)	(22.63/0.67)
Liver and pancreas	CHOL	***(24.70/0.66)***	**(26.30/0.74)**	(19.97/0.41)	(26.19/0.74)	(25.45/0.71)	(25.30/0.70)	(24.52/0.69)
	LIHC	(22.86/0.60)	**(24.26/0.69)**	(18.22/0.38)	**(24.25/0.70)**	(23.45/0.66)	(23.29/0.64)	(22.54/0.63)
	PAAD	(24.03/0.61)	**(25.47/0.70)**	(19.36/0.39)	(25.34/0.70)	(23.45/0.66)	(24.60/0.66)	(23.54/0.65)
Melanoma	SKCM	(22.67/0.66)	(24.56/0.76)	(17.78/0.42)	**(24.68/0.77)**	(23.67/0.73)	(23.21/0.70)	(22.83/0.72)
	UVM	(24.32/0.58)	**(25.39/0.66)**	(19.97/0.38)	(25.36/0.67)	(24.68/0.64)	(24.75/0.63)	(23.77/0.62)
Prostate and testis	PRAD	(23.90/0.59)	**(25.15/0.68)**	(19.42/0.38)	(25.08/0.69)	(24.41/0.66)	(24.34/0.64)	(23.42/0.63)
	TGCT	*(21.46/0.61)*	(23.02/0.72)	(17.64/0.44)	**(23.04/0.73)**	(22.00/0.68)	(22.00/0.66)	(21.35/0.67)
Pulmonary	LUAD	(23.25/0.63)	**(24.78/0.72)**	(18.37/0.39)	(23.04/0.73)	(23.93/0.69)	(23.77/0.67)	(22.99/0.67)
	LUSC	(23.12/0.62)	**(24.57/0.71)**	(18.42/0.40)	(24.48/0.71)	(23.67/0.68)	(23.62/0.67)	(22.81/0.66)
	MESO	(22.49/0.61)	**(24.01/0.71)**	(18.59/0.43)	**(23.97/0.72)**	(23.05/0.68)	(23.09/0.67)	(22.31/0.66)
Urinary tract	BLCA	(22.58/0.60)	**(24.02/0.70)**	(18.42/0.40)	**(23.98/0.71)**	(23.11/0.67)	(23.10/0.65)	(22.32/0.65)
	KIRC	(24.45/0.65)	**(25.96/0.73)**	(19.29/0.39)	**(25.93/0.74)**	(25.09/0.70)	(25.02/0.69)	(24.24/0.69)
	KIRP	(22.56/0.59)	**(23.99/0.69)**	(18.03/0.37)	(24.09/0.71)	(23.14/0.66)	(23.04/0.64)	(22.43/0.65)
	KICH	(23.79/0.64)	(25.18/0.72)	(18.37/0.38)	**(25.22/0.73)**	(24.47/0.70)	(24.12/0.67)	(23.54/0.69)

Another comparison is also provided in Table 2. In this table, the highest values with respect to the subtype are reported in bold. Two networks, DBPN and RCAN, mostly achieved the best results. The DBPN network is superior to others in 15 subtypes, while RCAN is also superior in 15 subtypes. If one network outperforms the other for one measure (SSIM or PSNR), both are mentioned in bold.

**Fig. 3:**
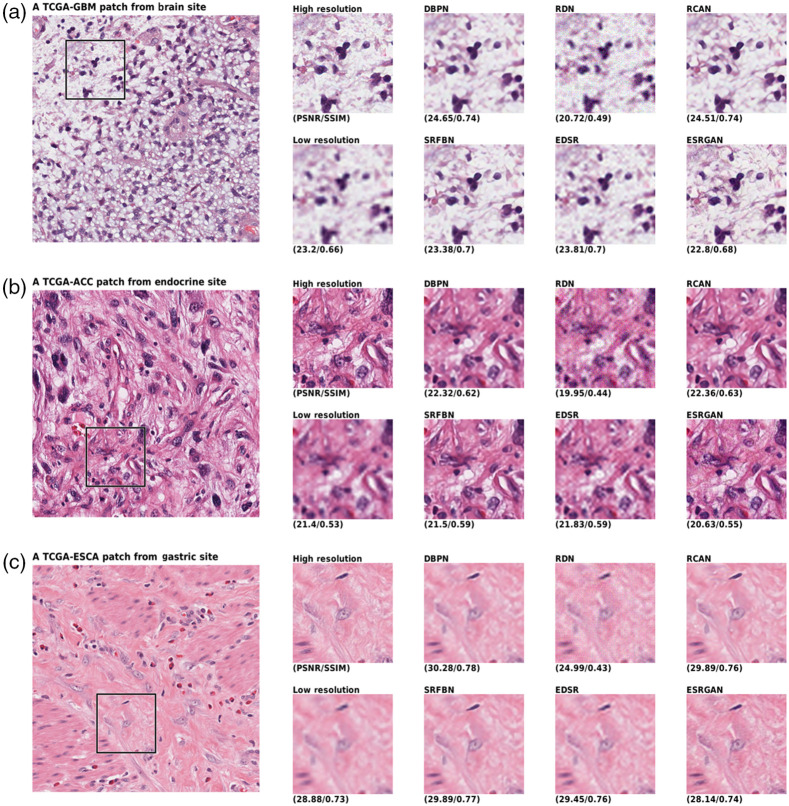
Qualitative results comparison of the six networks and the HR and LR images of (a) brain, (b) endocrine, and (c) gastric sites. The LR is fed to network to generate images and the large image on left shows the span box.

**Fig. 4:**
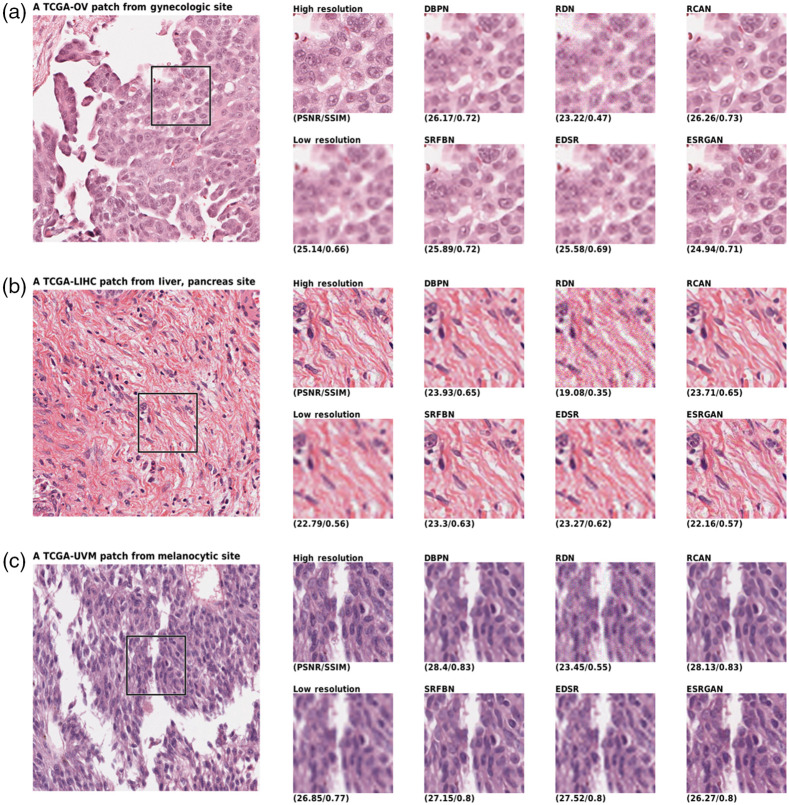
Qualitative results comparison of the six networks and the HR and LR of (a) gynecologic, (b) liver and pancreas, and (c) melocytic sites; The low resolution is fed to network to generate images and the large image on left shows the span box.

**Fig. 5:**
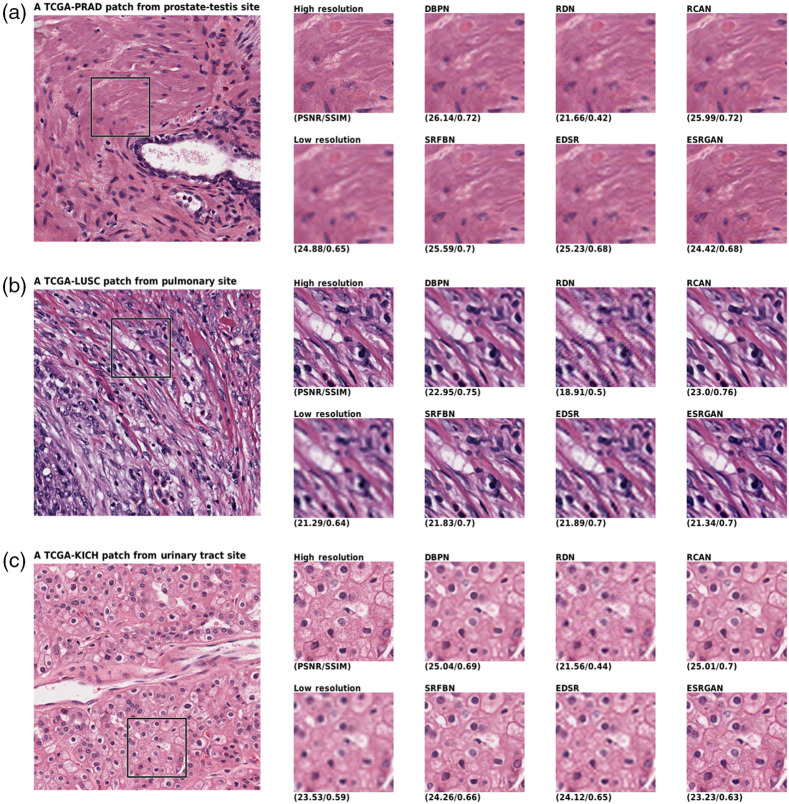
Qualitative results comparison of the six networks and the HR and LR of (a) prostate-testis, (b) pulmonary, and (c) urinary tract sites. The low resolution is fed to network to generate images and the large image on left shows the span box.

The qualitative comparison of the generated images shown in Figs. 3, 4, and 5. A randomly selected patch per site is shown in which another randomly selected window has been taken into focus. The window has been compared from eight approaches, including the HR, LR, and the six networks generation. The LR image shows the bicubic interpolation of the 4× downsampled image. Network inputs were the LR image, while the ideal outcome was a HR image. The PSNR and SSIM of the images are also given below. Taking a look at images, the ESRGAN-generated images look most like the HR image.

As shown in the figures, the networks can map the LR images to a reasonable HR image that seems to include a lot of details. This has been achieved due to the usage of GANs. Overall, ESRGAN provided the sharpest images in reconstructing the HR image, while RCAN and DBPN provided the closest estimation of unknown pixel values. Although ESRGAN images were sharp enough to clearly make the cells distinguishable, it could be observed that due to the generative nature of the processing, the formation of cells is slightly manipulated based on the training of the network. In contrast, RCAN and DBPN could produce more accurate cellular shapes. However, the edges were not as sharp as ESRGAN.

In addition, to evaluate the applicability of the SR to histopathology images, we performed two breast cancer-focused human assessment studies. A comprehensive evaluation by three board-certified pathologists was performed to determine the quality of these images. The SR images in this part are generated by the ESRGAN network. First, a diagnostic visual inspection has been conducted where pathologists were asked to classify images in eight categories. The categories (types) included breast tumors, benign and malignant, where each had four subtypes. The questions included four distinct histological categories of benign breast tumors: adenosis, fibroadenoma, phyllodes tumor, and tubular adenoma; and four malignant breast tumors: carcinoma, lobular carcinoma, mucinous carcinoma, and papillary carcinoma. Each pathologist evaluated 64 images, of which half were SR and the other half were HR (in random order). Second, we assessed the image quality preference of the pathologists by presenting them with 50 pairs of images, each consisting of the original image and the SR image. To prevent observer bias toward any of the image categories, the images were arranged at random order (e.g., HR and SR). The outcomes are shown in Table 3.

**Table 3:** Pathologist assessment of fake (SR) versus real (HR) images; type defines benign versus malignant; and subtype is the eight categories of BreakHis dataset.

	Diagnostic classification assessment results				
	SR patch		HR patch		
	Accuracy (%)	Kappa score	Accuracy (%)	Kappa score	
Pathologist one					
Type	78.1	0.56	75.0	0.50	
Subtype	15.6	0.04	21.9	0.11	
Pathologist two					
Type	78.1	0.56	71.9	0.44	
Subtype	31.2	0.21	18.7	0.07	
Pathologist three					
Type	71.9	0.44	81.3	0.62	
Subtype	25.0	0.14	34.4	0.25	
Average					
Type	76.0	0.52	76.0	0.52	
Subtype	24.0	0.13	25.0	0.14	
	**Quality preference results distribution (%)**				
	**Strong SR**	**Slight SR**	**No distinction**	**Slight HR**	**Strong HR**
Pathologist one	4.0	30.0	64.0	6.0	0.0
Pathologist two	6.0	26.0	14.0	48.0	6.0
Pathologist three	4.0	64.0	32.0	0.0	0.0
Average	4.7	40.0	36.7	18.0	2.0

The diagnostic findings reveal that there is no significant difference between the usage of generated SR images versus original HR images. This is demonstrated by the higher accuracy or kappa score of the evaluation diagnosis in six instances with HR pictures versus six cases involving SR images. As shown in Table 3, assessing SR images, the third pathologist received higher diagnostic score values. The second pathologist obtained higher diagnostic scores for HR pictures, but the first pathologist had slightly better findings (e.g., 3.1% higher malignancy detection accuracy) for type and subtype identification in SR and HR visuals, respectively.

Although this may be subject to higher observer variability, the image quality preference findings show that SR image are generally preferred by specialists. According to the data, just one pathologist (i.e., number two) favored the HR images, and even in this instance, the expert prioritized or was unable to identify substantial differences in 46% of the images. One pathologist regarded 64% of the images to be fairly comparable, although in 34% of the instances all observers preferred the SR images. Ultimately, the second pathologist deemed 68% of SR photos to be preferable, whereas the rest of the images were found without discernible difference.

In summary, the gigapixel nature of WSI in digital pathology is absolute necessary to generate HR images for diagnostic purposes. However, the large size of WSIs also creates obstacles for practical utilities of digital pathology, most notable extreme demands for high-performance storage. This study, based on the largest publicly available dataset, demonstrated that the deep GANs are indeed capable of predicting HR images from their LR versions. Some generative models may be more suitable for computerized processing (e.g., RCAN and DBPN), and some other based on adversarial training for visual inspection (e.g., ESRGAN). Our findings indicate that actual/real (HR) and synthetic/fake (SR) images are identified by pathologists at equivalent accuracy levels. As well, the pathologists have even visually preferred synthetic images to the real images in many cases.

## References

[1] Eadie L. H. Taylor P. Gibson A. P., “A systematic review of computer-assisted diagnosis in diagnostic cancer imaging,” Eur. J. Radiol. 81(1), e70–e76 (2012). EJRADR 0720-048X 10.1016/j.ejrad.2011.01.098 21345631

[2] Niazi M. K. K. Parwani A. V. Gurcan M. N., “Digital pathology and artificial intelligence,” Lancet Oncol. 20(5), e253–e261 (2019). LOANBN 1470-2045 10.1016/S1470-2045(19)30154-8 31044723 PMC8711251

[3] Fu Y. et al., “Deep learning in medical image registration: a review,” Phys. Med. Biol. 65(20), 20TR01 (2020). PHMBA7 0031-9155 10.1088/1361-6560/ab843e PMC7759388 32217829

[4] Razzak M. I. Naz S. Zaib A., “Deep learning for medical image processing: overview, challenges and the future,” in Classification in BioApps, pp. 323–350 (2018).

[5] Srinidhi C. L. Ciga O. Martel A. L., “Deep neural network models for computational histopathology: a survey,” Med. Image Anal. 67, 101813 (2020). 10.1016/j.media.2020.101813 33049577 PMC7725956

[6] Tao X. et al., “Detail-revealing deep video super-resolution,” in Proc. IEEE Int. Conf. Comput. Vision, pp. 4472–4480 (2017).

[7] Van Ouwerkerk J., “Image super-resolution survey,” Image Vision Comput. 24(10), 1039–1052 (2006). IVCODK 0262-8856 10.1016/j.imavis.2006.02.026

[8] Yang W. et al., “Deep learning for single image super-resolution: a brief review,” IEEE Trans. Multimedia 21(12), 3106–3121 (2019). 10.1109/TMM.2019.2919431

[9] Christensen-Jeffries K. et al., “Super-resolution ultrasound imaging,” Ultrasound Med. Biol. 46(4), 865–891 (2020). USMBA3 0301-5629 10.1016/j.ultrasmedbio.2019.11.013 31973952 PMC8388823

[r10] Greenspan H., “Super-resolution in medical imaging,” Comput. J. 52(1), 43–63 (2009). 10.1093/comjnl/bxm075

[r11] Rousseau F. et al., “On super-resolution for fetal brain MRI,” Lect. Notes Cmput. Sci. 6362, 355–362 (2010). 10.1007/978-3-642-15745-5_44 PMC3319126 20879335

[12] Van Reeth E. et al., “Super-resolution in magnetic resonance imaging: a review,” Concepts Magn. Reson. Part A 40(6), 306–325 (2012). 10.1002/cmr.a.21249

[13] Zhang L. et al., “A super-resolution reconstruction algorithm for surveillance images,” Signal Process. 90(3), 848–859 (2010). SPRODR 0165-1684 10.1016/j.sigpro.2009.09.002

[r14] Seibel H. Goldenstein S. Rocha A., “Eyes on the target: Super-resolution and license-plate recognition in low-quality surveillance videos,” IEEE Access 5, 20020–20035 (2017). 10.1109/ACCESS.2017.2737418

[15] Shamsolmoali P. et al., “Deep convolution network for surveillance records super-resolution,” Multimedia Tools Appl. 78(17), 23815–23829 (2019). 10.1007/s11042-018-5915-7

[16] Dai D. et al., “Is image super-resolution helpful for other vision tasks?,” in IEEE Winter Conf. Appl. of Comput. Vision (WACV), IEEE, pp. 1–9 (2016). 10.1109/WACV.2016.7477613

[r17] Haris M. Shakhnarovich G. Ukita N., “Task-driven super resolution: Object detection in low-resolution images,” arXiv:1803.11316 (2018).

[18] Sajjadi M. S. Scholkopf B. Hirsch M., “Enhancenet: Single image super-resolution through automated texture synthesis,” in Proc. IEEE Int. Conf. Comput. Vision, pp. 4491–4500 (2017).

[19] Keys R., “Cubic convolution interpolation for digital image processing,” IEEE Trans. Acoust. Speech Signal Process. 29(6), 1153–1160 (1981). IETABA 0096-3518 10.1109/TASSP.1981.1163711

[20] Duchon C. E., “Lanczos filtering in one and two dimensions,” J. Appl. Meteorol. Climatol. 18(8), 1016–1022 (1979). 10.1175/1520-0450(1979)018<1016:LFIOAT>2.0.CO;2

[21] Sun J. Shum H.-Y., “Image super-resolution using gradient profile prior,” US Patent 9,064,476 (2015).

[22] Kim K. I. Kwon Y., “Single-image super-resolution using sparse regression and natural image prior,” IEEE Trans. Pattern Anal. Mach. Intell. 32(6), 1127–1133 (2010). ITPIDJ 0162-8828 10.1109/TPAMI.2010.25 20431136

[23] Freeman W. T. Jones T. R. Pasztor E. C., “Example-based super-resolution,” IEEE Comput. Graphics Appl. 22(2), 56–65 (2002). ICGADZ 0272-1716 10.1109/38.988747

[24] Yang J. et al., “Image super-resolution as sparse representation of raw image patches,” in IEEE Conf. Comput. Vision and Pattern Recognit., IEEE, pp. 1–8 (2008). 10.1109/CVPR.2008.4587647

[25] Martin D. et al., “A database of human segmented natural images and its application to evaluating segmentation algorithms and measuring ecological statistics,” in Proc. 8th Int. Conf. Comput. Vision, Vol. 2, pp. 416–423 (2001).

[r26] Agustsson E. Timofte R., “Ntire 2017 challenge on single image super-resolution: Dataset and study,” in Proc. IEEE Conf. Comput. Vision and Pattern Recognit. Workshops, pp. 126–135 (2017). 10.1109/CVPRW.2017.150

[r27] Dong C. Loy C. C. Tang X., “Accelerating the super-resolution convolutional neural network,” Lect. Notes Comput. Sci. 9906, 391–407 (2016). LNCSD9 0302-9743 10.1007/978-3-319-46475-6_25

[28] Matsui Y. Yamasaki T. Aizawa K., “Interactive manga retargeting,” in ACM SIGGRAPH 2011 Posters, SIGGRAPH ’11, Association for Computing Machinery, New York (2011).

[29] Dong C. et al., “Image super-resolution using deep convolutional networks,” IEEE Trans. Pattern Anal. Mach. Intell. 38(2), 295–307 (2015). ITPIDJ 0162-8828 10.1109/TPAMI.2015.2439281 26761735

[30] Ledig C. et al., “Photo-realistic single image super-resolution using a generative adversarial network,” in Proc. IEEE Conf. Comput. Vision and Pattern Recognit., pp. 4681–4690 (2017).

[31] Kim J. Kwon Lee J. Mu Lee K., “Accurate image super-resolution using very deep convolutional networks,” in Proc. IEEE Conf. Comput. Vision and Pattern Recognit., pp. 1646–1654 (2016). 10.1109/CVPR.2016.182

[r32] Lai W.-S. et al., “Deep laplacian pyramid networks for fast and accurate super-resolution,” in Proc. IEEE Conf. Comput. Vision and Pattern Recognit., pp. 624–632 (2017). 10.1109/CVPR.2017.618

[33] Ahn N. Kang B. Sohn K.-A., “Fast, accurate, and lightweight super-resolution with cascading residual network,” in Proc. Eur. Conf. Comput. Vision (ECCV), pp. 252–268 (2018).

[34] Johnson J. Alahi A. Fei-Fei L., “Perceptual losses for real-time style transfer and super-resolution,” Lect. Notes Comput. Sci. 9906, 694–711 (2016). LNCSD9 0302-9743 10.1007/978-3-319-46475-6_43

[35] Bulat A. Tzimiropoulos G., “Super-FAN: integrated facial landmark localization and super-resolution of real-world low resolution faces in arbitrary poses with GANs,” in Proc. IEEE Conf. Comput. Vision and Pattern Recognit., pp. 109–117 (2018). 10.1109/CVPR.2018.00019

[36] Lim B. et al., “Enhanced deep residual networks for single image super-resolution,” in Proc. IEEE Conf. Comput. Vision and Pattern Recognit. Workshops, pp. 136–144 (2017). 10.1109/CVPRW.2017.151

[37] Wang Y. et al., “A fully progressive approach to single-image super-resolution,” in Proc. IEEE Conf. Comput. Vision and Pattern Recognit. Workshops, pp. 864–873 (2018).

[38] Lyu Q. et al., “Super-resolution MRI through deep learning,” arXiv:1810.06776 (2018).

[39] Mukherjee L. et al., “Convolutional neural networks for whole slide image superresolution,” Biomed. Opt. Express 9(11), 5368–5386 (2018). BOEICL 2156-7085 10.1364/BOE.9.005368 30460134 PMC6238924

[40] Mukherjee L. et al., “Super-resolution recurrent convolutional neural networks for learning with multi-resolution whole slide images,” J. Biomed. Opt. 24(12), 126003 (2019). JBOPFO 1083-3668 10.1117/1.JBO.24.12.126003 31837128 PMC6910074

[41] Lin W. Dong L., “Adaptive downsampling to improve image compression at low bit rates,” IEEE Trans. Image Process. 15(9), 2513–2521 (2006). IIPRE4 1057-7149 10.1109/TIP.2006.877415 16948298

[42] Irani M. Peleg S., “Improving resolution by image registration,” CVGIP, Graphics Models Image Process. 53(3), 231–239 (1991). CGMPE5 1049-9652 10.1016/1049-9652(91)90045-L

[43] Wang Z. Chen J. Hoi S. C., “Deep learning for image super-resolution: a survey,” IEEE Trans. Pattern Anal. Mach. Intell. 43, 3365–3387 (2020). ITPIDJ 0162-8828 10.1109/TPAMI.2020.2982166 32217470

[44] Zhang K. Zuo W. Zhang L., “Learning a single convolutional super-resolution network for multiple degradations,” in Proc. IEEE Conf. Comput. Vision and Pattern Recognit., pp. 3262–3271 (2018).

[45] Carlson R. E. Fritsch F. N., “Monotone piecewise bicubic interpolation,” SIAM J. Numer. Anal. 22(2), 386–400 (1985). SJNAEQ 0036-1429 10.1137/0722023

[46] Zhang Y. et al., “Image super-resolution using very deep residual channel attention networks,” in Proc. Eur. Conf. Comput. Vision (ECCV), pp. 286–301 (2018).

[47] Karras T. Laine S. Aila T., “A style-based generator architecture for generative adversarial networks,” in IEEE Conf. Comput. Vision and Pattern Recognit. (CVPR) (2019). 10.1109/CVPR.2019.00453 32012000

[48] Li Z. et al., “Feedback network for image super-resolution,” in Proc. IEEE/CVF Conf. Comput. Vision and Pattern Recognit., pp. 3867–3876 (2019).

[49] Krizhevsky A. Sutskever I. Hinton G. E., “Imagenet classification with deep convolutional neural networks,” in Adv. Neural Inf. Process. Syst. 25, pp. 1097–1105 (2012).

[50] Haris M. Shakhnarovich G. Ukita N., “Deep back-projection networks for super-resolution,” in Proc. IEEE Conf. Comput. Vision and Pattern Recognit., pp. 1664–1673 (2018).

[51] Zhang Y. et al., “Residual dense network for image super-resolution,” in IEEE Conf. Comput. Vision and Pattern Recognit. (CVPR) (2018). 10.1109/CVPR.2018.00262

[52] Wang X. et al., “ESRGAN: enhanced super-resolution generative adversarial networks,” Lect. Notes Comput. Sci. 11133, 63–79 (2018). LNCSD9 0302-9743 10.1007/978-3-030-11021-5_5

[53] The Cancer Genome Atlas Program, 2008, https://www.cancer.gov/tcga (2023).

[54] Weinstein J. N. et al., “The cancer genome atlas pan-cancer analysis project,” Nat. Genet. 45(10), 1113 (2013). NGENEC 1061-4036 10.1038/ng.2764 24071849 PMC3919969

[55] Spanhol F. A. et al., “A dataset for breast cancer histopathological image classification,” IEEE Trans. Biomed. Eng. 63(7), 1455–1462 (2015). IEBEAX 0018-9294 10.1109/TBME.2015.2496264 26540668

